# Magnetic Detection of Cancer Cells Using Tumor-Homing Peptide-Modified Magnetic Nanoparticles

**DOI:** 10.3390/bios16010045

**Published:** 2026-01-05

**Authors:** Shengli Zhou, Yuji Furutani, Kei Yamashita, Sakuya Kako, Kazunori Watanabe, Toshihiko Kiwa, Takashi Ohtsuki

**Affiliations:** Department of Interdisciplinary Science and Engineering in Health Systems, Okayama University, 3-1-1 Tsushimanaka, Okayama 700-8530, Japan; p0ux6145@s.okayama-u.ac.jp (S.Z.); p0z80rhm@s.okayama-u.ac.jp (Y.F.); k_watanabe@okayama-u.ac.jp (K.W.); kiwa@ec.okayama-u.ac.jp (T.K.)

**Keywords:** magnetic nanoparticle, tumor-homing peptide, superconducting quantum interference devices

## Abstract

Magnetic nanoparticles (MNPs) provide a platform for target detection because of their magnetic responsiveness to alternating magnetic fields (AMFs). We developed a detection method using MNPs modified with tumor-homing peptides (THPs), PL1 and PL3, which selectively bind to protein components enriched in malignant tissues. THP-MNPs were synthesized using maleimide-PEG-NHS linkers and characterized using transmission electron microscopy. Human glioblastoma cancer U87MG and normal tissue-derived HEK293 cells were incubated with THP-MNPs, and the magnetic signals were measured using a high-temperature superconducting quantum interference device (SQUID) magnetometer under an AMF (1.06 kHz). Dark-field microscopy confirmed the preferential binding of THP-MNPs to U87MG cells. In the absence of cells, THP-MNPs exhibited AMF-dependent signal enhancement, which correlated with particle size reduction due to THP release. This increase was completely suppressed in the presence of U87MG cells, indicating a strong THP-mediated interaction. PL3-MNPs exhibited superior discrimination between malignant and non-malignant cells. These results demonstrate that SQUID-based magnetic measurements using THP-MNPs enable rapid and label-free cancer cell detection.

## 1. Introduction

Magnetic nanoparticles (MNPs) made of iron oxides are widely used as biosensors and diagnostic platforms to detect viruses, microorganisms, and tumors [1,2]. Two strategies have been reported for the detection of specific cells using MNPs. The first strategy uses a magnet to collect MNPs that capture target cells. Magnetically captured cells are detected using non-magnetic methods, such as surface-enhanced Raman scattering and immunostaining [3,4]. The second strategy involves the detection of the magnetic responses of MNPs. For example, circulating tumor cells have been detected using MNPs modified with target-binding antibodies by monitoring their magnetic responses [5]. Magnetic resonance imaging, based on nuclear magnetic resonance, detects cancer using magnetic signals in combination with MNPs and is generally used for solid tumors [6].

This study focused on a strategy to detect target cells using the magnetic response of MNPs under an alternating magnetic field (AMF). MNPs are highly stable against AMF and harsh environments and do not photobleach like fluorescent dyes [7]. Bioassay methods that directly measure the magnetic responses from MNPs are simple and do not require purification or washing. Owing to their rapidity and simplicity, MNP magnetic response-based bioassays are attracting attention as potential testing tools in various fields, such as medical diagnosis and food safety [8,9,10,11].

The magnetic response of MNPs is based on the alternating current (AC) magnetic susceptibility, χ, which is generally described by the Debye model and is expressed as

$$\chi =\frac{\chi_{0}}{1-i2\pi f_{ac}\tau_{eff}},$$

where $\tau_{eff}$ indicates effective relaxation time, $\chi_{0}$ is static susceptibility, and $f_{ac}$ is the frequency of an applied ac magnetic field. In a liquid, the magnetic moments of MNPs relax through two independent mechanisms: Brownian and Néel relaxations. The Néel relaxation time depends on the magnetic core volume of the MNPs, while the Brownian relaxation time is proportional to the hydrodynamic particle volume. For MNPs with a core diameter larger than approximately 30 nm, the Néel relaxation time becomes much shorter than 1 s, indicating that Brownian relaxation dominates the effective relaxation process. Consequently, the observed AC magnetic susceptibility of such particles is mainly governed by Brownian relaxation.

A superconducting quantum interference device (SQUID) is a highly sensitive instrument used to detect the magnetic response of MNPs [12,13]. SQUIDs have been used with MNPs to detect cells, such as HER2-expressing cells [14] and bacteria [15]. Among these approaches, antibody-modified MNPs show high specificity, and their detection using SQUIDs has achieved high sensitivity [12,16]. Despite these efforts, cell detection using SQUID with MNPs modified with non-antibody-targeting molecules has not yet been reported.

A common strategy for detecting cancer cells using MNPs is to modify them with cancer cell recognition molecules. The modification of MNPs with anti-HER2 antibodies resulted in enhanced internalization by cancer cells [17]. MNPs modified with pH (low)-insertion peptides have been shown to accumulate efficiently in solid tumors [18]. Similarly, the modification of MNPs with folic acid resulted in increased uptake by folate receptor-positive cancer cells, such as HNE-19 [19].

In this study, we aimed to demonstrate the magnetic detection of cancer cells using MNPs modified with tumor-homing peptides (THPs), PL1 and PL3. PL1 has a high affinity for the C-domain of tenascin-C (TNC-C) and extra domain-B (ED-B) of fibronectin, which are prominently enriched in malignant tissues, thereby enabling selective recognition of cancer cells [20]. PL3 targets TNC-C and engages neuropilin-1 through its CendR motif, conferring enhanced tumor selectivity and preferential binding to cancer cells [21]. In this study, we attempted to detect cancer cells using PL1-MNPs and PL3-MNPs. We examined the differences in the affinities of PL1 and PL3 for normal tissue-derived HEK293 and glioblastoma U87MG cells. We used our custom-made SQUID system to measure the changes in the magnetic signals under AMF.

## 2. Materials and Methods

### 2.1. Preparation of THP-Modified MNPs

**Figure 1:**
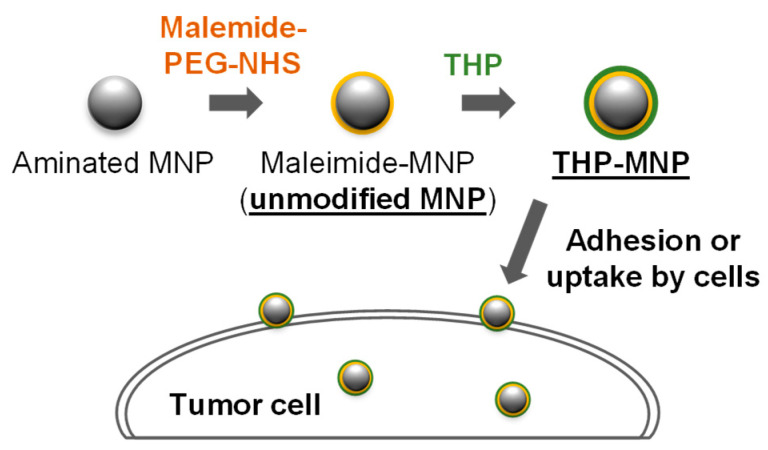
Schematic diagram of the synthesis of tumor-homing peptide (THP)-magnetic nanoparticles (MNPs) and their interactions with tumor cells.

THP-modified MNPs were prepared according to the method described by Zhou et al. [22]. Briefly, the aminated MNPs Synomag^®︎^-D50 (50 nm containing 30 nm maghemite core) (Micromod Partikeltechnologie GmbH, Rostock, Germany) were first maleimide-modified using maleimide-2K-polyethylene glycol-N-hydroxysuccinimide ester (maleimide-PEG-NHS) (Biopharma PEG Scientific, Watertown, NY, USA). This is referred to as “unmodified MNPs.” THPs were coupled to MNP via a reaction with maleimide groups via the thiol groups of the THPs (Figure 1). We used PL1 (sequence: PPRRGLIKLKTSWGC; the C-terminal WGC sequence was added to the original PL1 sequence). WG is a linker, C includes a thiol group for the reaction with maleimide) and PL3 (sequence: CLAWAGRGRLVR; the N-terminal CLAW sequence was added to the original PL3 sequence as a linker and reactive group). These peptides were prepared by Fmoc-based solid-phase peptide synthesis and provided by the Central Research Laboratory of Okayama University Medical School.

### 2.2. Cell Culture

All cells were cultured at 37 °C in a 5% CO_2_ atmosphere. HEK293 (PEAKrapid) cells obtained from the American Type Culture Collection (ATCC) (Manassas, VA, USA) were cultured in high-glucose Dulbecco’s modified Eagle medium (D-MEM) (Fujifilm, Tokyo, Japan) containing 10% fetal bovine serum (FBS) (Sigma-Aldrich, Muskegon, MI, USA), 100 U/mL penicillin, and 100 μg/mL streptomycin (Gibco, Carlsbad, CA, USA). U87MG cells obtained from ATCC were cultured in E-MEM medium (Fujifilm) with 10% FBS, 1% of 100× nonessential amino acid solution (Fujifilm), 1 mM sodium pyruvate (Thermo Fisher Scientific, Tokyo, Japan), 100 U/mL penicillin, and 100 μg/mL streptomycin.

### 2.3. Dark-Field Microscopy

U87MG and HEK293 cells were seeded in 2 mL of E-MEM or RPMI1640 medium in a 12-well plate with a cover glass (2 × 10^5^ cells/well). After 24 h of incubation, a 200 μg/mL MNP suspension was added along with T buffer (20 mM HEPES-KOH [pH 7.6], 115 mM NaCl, 5.4 mM KCl, 1.8 mM CaCl_2_, 0.8 mM MgCl_2_, and 13.8 mM glucose), and the mixture was incubated at 37 °C for 1 h. Next, the cells were washed three times with 1× PBS to remove the MNPs that did not bind to the cells. MNPs binding to cells were visualized using dark-field microscopy (CX43 microscope; Olympus, Tokyo, Japan).

### 2.4. Magnetic Measurements of THP-Modified MNPs with Cells

**Figure 2:**
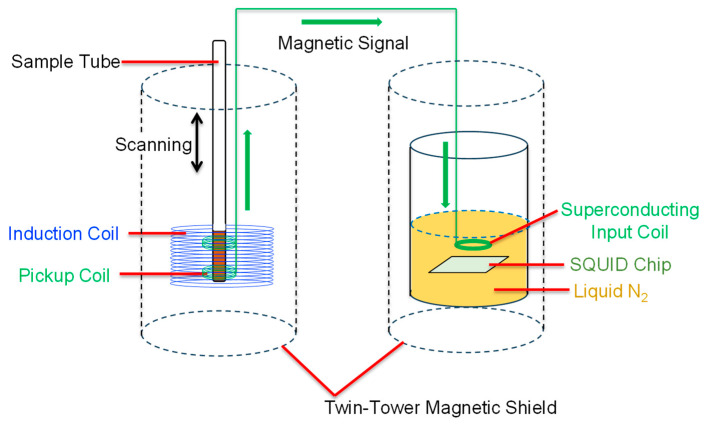
HTS-SQUID AC magnetometer used in this study.

The high-temperature superconducting (HTS)-SQUID AC magnetometer was used to evaluate the magnetic signals of the MNPs (Figure 2). Details of the developed system have been previously reported [13,23]. For the measurements, the cells were treated with 0.25 *w*/*v*% Trypsin-1 mmol/L ethylenediaminetetraacetic acid (EDTA)-4Na solution (Fujifilm), centrifuged at 200× *g*, and suspended in T buffer. THP-modified MNPs (MNP concentration of 200 µg/mL) were then mixed with the cell suspension (U87MG or HEK293, 1 × 10^5^ cells/mL). The sample (5 × 10^3^ cells in 50 μL) was placed in a microglass sample tube and then inserted into an excitation coil, and an AMF with a frequency of 1.06 kHz and a magnetic field of 8 mTpp was applied. To eliminate the influence of diamagnetic components, a third-harmonic signal was detected using a lock-in technique. In addition, the sample was moved up and down ten times at a speed of 4 cm/s to reduce the baseline drift of the magnetic signal, and its peak-to-peak averaged magnetic signal was used to evaluate the MNP signal.

### 2.5. Transmission Electron Microscopy (TEM)

The THP-MNPs were imaged using an H-7650 transmission electron microscope (Hitachi, Tokyo, Japan) with 200 µg/mL of THP-MNPs dissolved in T buffer.

## 3. Results and Discussion

### 3.1. Attachment and Uptake of MNPs to Cells

**Figure 3:**
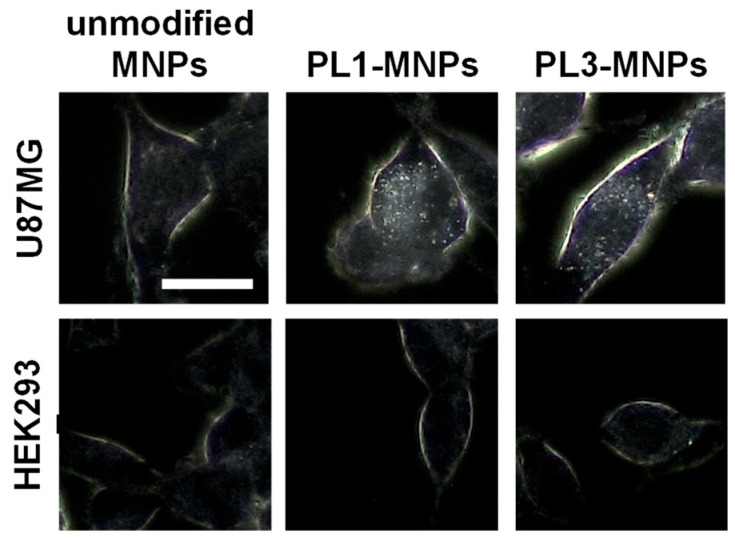
Dark-field images of HEK293 and U87MG cells with MNPs. Scale bar indicates 20 µm.

THP-MNPs were prepared, and their interactions with tumor cells were investigated. Dark-field microscopy was used to image the binding of MNPs to the cells. As shown in Figure 3, PL1-MNPs and PL3-MNPs bound more efficiently to U87MG tumor cells than unmodified MNPs. In contrast, THP-modified and unmodified MNPs barely bound to HEK293 cells derived from normal tissues. The cell periphery was slightly visible under a dark-field microscope, even without MNPs. However, when U87MG cells were mixed with THP-MNPs, the cell periphery glowed more brightly, and MNPs (dots) were visible within the cells.

### 3.2. Magnetic Signal Intensity of THP-Modified MNPs Increased with Continuous AMF Irradiation

**Figure 4:**
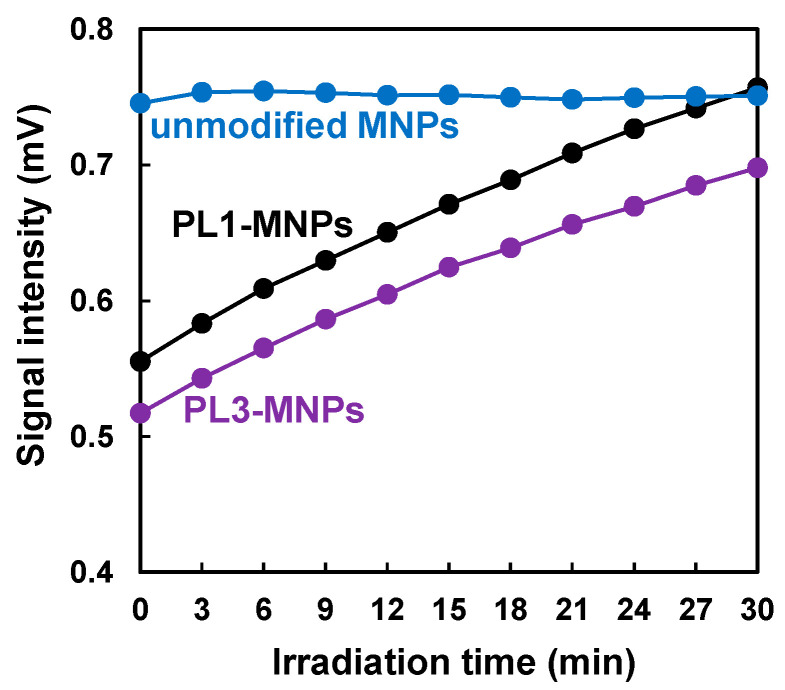
Magnetic signal intensities of MNPs under AMF irradiation measured using the HTS-SQUID system.

Magnetic measurements were initially performed using MNPs without cells. The magnetic signal intensity of the unmodified MNPs remained constant during AMF irradiation (Figure 4). In contrast, the magnetic signal intensities of the MNPs modified with PL1 and PL3 gradually increased under AMF and reached intensities similar to those of the unmodified MNPs after 30 min of irradiation. As the metal (Fe_2_O_3_) core size of these MNPs was 30 nm, the magnetic signal intensity under a 1.06 kHz AMF was considered to be dependent on Brownian relaxation [24]. Based on Brownian relaxation, the smaller the particle size, the higher the magnetic signal intensity. Therefore, the gradual increase in the magnetic signal intensity (Figure 4) suggests that the sizes of the PL1- and PL3-MNPs decreased under AMF irradiation.

### 3.3. AMF Irradiation Decreased the Size of THP-Modified MNPs

**Figure 5:**
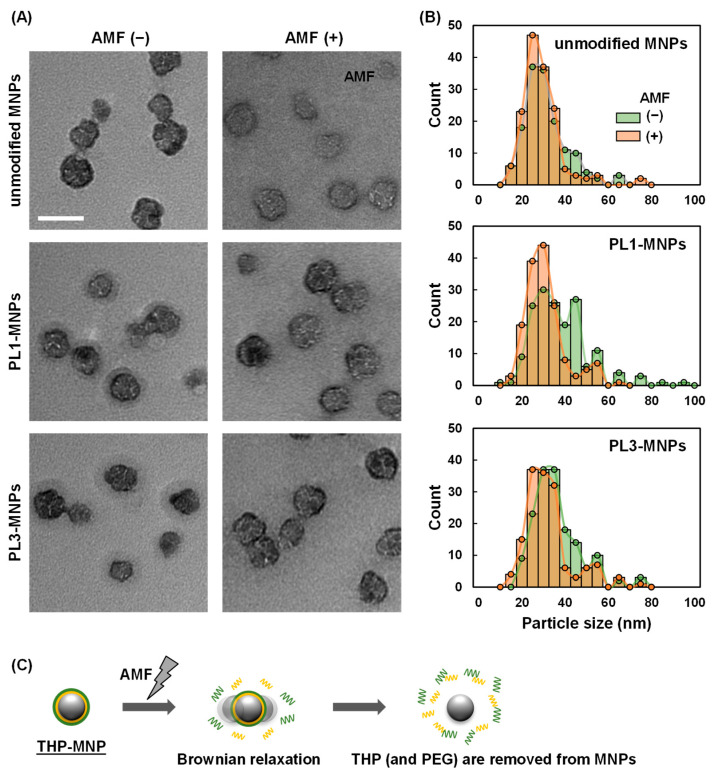
Transmission electron microscopy (TEM) images (**A**) and particle size distributions of MNPs (**B**) before and after alternating magnetic field (AMF) irradiation. Scale bar indicates 100 nm. (**C**) proposed mechanism for the decrease in THP-MNPs’ size during AMP irradiation.

According to the TEM images, the THP-modified MNPs had a layer of organic material-like shells on the outside of the metal core (Figure 5A). In contrast, the unmodified MNPs did not have a shell layer, indicating that the shell layer was composed of THPs. After AMF irradiation, the shells of the THP-MNPs became invisible, similar to those of unmodified MNPs. The average particle size of the THP-MNPs decreased after AMF irradiation (Figure 5B, Table 1). The larger particle size of the THP-MNPs compared to that of the unmodified MNPs was related to the low magnetic signal intensity of the THP-modified MNPs before AMF irradiation (time = 0), as shown in Figure 4. The decrease in the size of PL1- and PL3-MNPs after AMF irradiation (Table 1) is consistent with our speculation that the increase in the magnetic signal intensity under AMF irradiation (Figure 4) is related to the decrease in the size of THP-MNPs. Particularly, the reduction in particle size of PL1-MNPs was statistically significant (Figure S1).

Although the reason for the size reduction after AMF irradiation is unclear, Brownian relaxation may be the cause. According to Izak-Nau et al. [25], Brownian relaxation can cause MNPs to tumble and shake, resulting in the release of polymers linked to PEG. In our THP-MNPs, THPs were connected to MNPs via PEG linkers. Additionally, our magnetic measurement conditions (1.06 kHz, 30 nm metal core) are considered to cause relaxation, mainly due to Brownian relaxation. Therefore, similar to the results reported by Izak-Nau et al., Brownian relaxation may have caused the release of THPs from THP-MNPs (Figure 5C).

### 3.4. Increase in AMF-Dependent Magnetic Signal of THP-Modified MNPs Was Inhibited in the Presence of Tumor Cells

**Figure 6:**
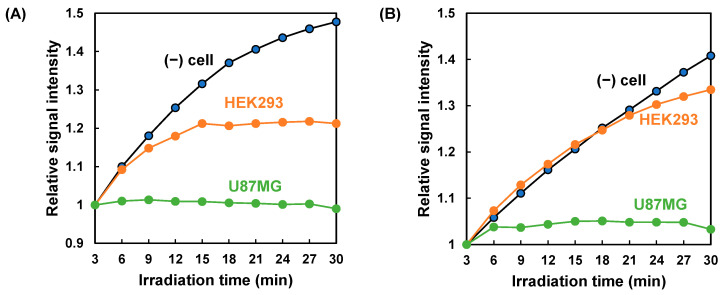
Magnetic signal intensities of (**A**) PL1-MNPs and (**B**) PL3-MNPs that reacted with HEK293 and U87MG cell suspensions. The signal intensities were normalized to the intensity at the start of the measurement.

In the absence of cells, the magnetic signal of THP-MNPs increased during the AMF irradiation (Figure 4). We investigated the magnetic signals of the THP-MNPs in the presence of cells. In contrast to the absence of cells, the increase in the magnetic signal of the PL1-MNPs induced by the AMF was completely suppressed in the presence of U87MG cells (Figure 6A). In the presence of HEK293 cells, the increase in the magnetic signal of the PL1-MNPs was moderately suppressed. When using PL3-MNPs, results similar to those of the PL1-MNPs were observed (Figure 6B); the magnetic signal of PL3-MNPs increased considerably in the absence of cells and the magnetic signal increase was almost completely inhibited in the presence of U87MG cells and slightly suppressed in the presence of HEK293 cells. These results indicate that PL1- and PL3-modified MNPs interacted more strongly with U87MG tumor cells than with non-malignant HEK293 cells. The AMF-induced increase in the THP-MNP magnetic signals in the absence of cells appeared to result from a decrease in particle size due to THP detachment (Figure 5). Considering this, the lack of change in the magnetic signal in the presence of U87MG cells suggests that these cells protected the THP-MNPs from degradation. Magnetic measurements using THP-MNPs and the HTS-SQUID system enabled us to distinguish between the presence and absence of U87MG tumor cells. PL3-MNP distinguished between HEK293 and U87MG cells more clearly than PL1-MNP did. PL3-MNP was able to detect U87MG cells at even lower concentrations than in the Figure 6 experiment (Figure S2).

This study was conducted using only glioblastoma cancer U87MG cells and normal tissue-derived HEK293 benign cells; however, the following points may be considered regarding the specificity of this method. PL1 targets TNC-C and fibronectin ED-B. PL3 targets TNC-C and engages neuropilin-1. TNC and neuropilin-1 are highly expressed in various cancer cells and tissues [26,27]. The fibronectin ED-B is highly expressed in human solid tumors [28]. The higher mRNA expression levels of TNC and neuropilin-1 in U87MG cells compared to HEK293 cells [26] is consistent with our results (higher accumulation of PL1-MNPs and PL3-MNPs in U87MG cells compared to HEK293 cells). Thus, PL1- and PL3-MNPs are considered capable of detecting various cancer cells.

## 4. Conclusions

In this study, we evaluated a method for detecting cancer cells by measuring the magnetic signal of THP-MNPs in a cell suspension exposed to AMF. Before AMF irradiation, the magnetic signal of the THP-MNPs was weaker than that of the unmodified MNPs. However, after AMF irradiation, the magnetic signal of the THP-MNPs increased to a level comparable to that of the unmodified MNPs. This suggests that despite their larger size, AMF irradiation causes THP detachment, reducing the size of the THP-MNPs to a level comparable to that of unmodified MNPs. This suggestion regarding the change in particle size was supported by TEM images. Mixing THP-MNPs with U87MG tumor cells completely suppressed the increase in the magnetic signal under the AMF. In contrast, when combined with non-malignant HEK293 cells, the degree of suppression was lower. When PL3-MNPs were used with HEK293 cells, the increase in the magnetic signal under the AMF was nearly identical to that observed in the absence of cells. These results demonstrate that magnetic measurements using THP-MNPs enable the detection of tumor cells in a solution. Unfortunately, neither PL1- nor PL3-MNPs showed an increase in signal intensity in a serum-containing medium under AMF irradiation, and thus cancer cells cannot be detected in the presence of serum. This is likely due to various components in the serum, such as proteins, adsorbing onto the THP-MNPs and hindering the desorption of THP from the MNPs. When detecting CTCs using this method, it would be necessary to remove the supernatant from the blood samples by centrifugation and resuspend the cells in a buffer.

PL1 is bi-specific to oncofetal fibronectin and TNC-C, which are nearly absent in the extracellular matrix of normal adult tissues but are upregulated in malignant tissues [29]. These target molecules are both components of the extracellular matrix but are suggested to exist on the cell surface via integrin binding [30,31]. PL3 targets TNC-C and the cell surface receptor, neuropilin-1 [21]. These cell surface target molecules likely facilitate the binding and uptake of PL1- and PL3-MNPs by the U87MG tumor cells. Because these target molecules are highly expressed in various cancer cells compared to non-cancerous cells, PL1- and PL3-MNPs are considered capable of detecting various types of cancer cells. Owing to its ability to detect tumor cells simply by mixing with MNPs without requiring a washing step, this technology is promising for cancer diagnosis, such as the detection of circulating tumor cells in blood.

HTS-SQUIDs have been used for medical diagnoses because they allow for more compact, cost-effective systems than low-temperature superconducting SQUIDs [32]. Therefore, our system has strong potential to be introduced into clinical settings in the near future.

## Figures and Tables

**Table 1:** Average particle sizes (±SEM) of the magnetic nanoparticles (MNPs) before and after alternating magnetic field (AMF) irradiation, determined from transmission electron microscopy (TEM) images. More than 150 particles were measured for each MNPs.

Particles	AMF	Particle Size (nm)
Unmodified MNPs	-	34.9 ± 12.6
PL1-MNPs	-	41.2 ± 14.0
PL3-MNPs	-	39.4 ± 11.8
Unmodified MNPs	+	32.5 ± 10.4
PL1-MNPs	+	34.8 ± 11.3
PL3-MNPs	+	36.4 ± 12.5

## Data Availability

The original contributions presented in this study are included in the article/Supplementary Materials. Further inquiries can be directed to the corresponding author.

## Supplementary Materials

The following supporting information can be downloaded at: https://www.mdpi.com/article/10.3390/bios16010045/s1, Figure S1. Average particle sizes (±SEM) of the magnetic nanoparticles (MNPs) before and after alternating magnetic field (AMF) irradiation, determined from transmission electron microscopy (TEM) images. More than 150 particles were measured for each MNPs. These results are expressed as means ± SEM. Statistical significance was analyzed using R [33] and EZR [34] software and determined using a two-way ANOVA with Tukey’s test. * *p* < 0.01, ** *p* < 0.001, ns: not significant. Figure S2. Magnetic signal intensities of PL3-MNPs that reacted with U87MG cell suspensions. The signal intensities were normalized to the intensity at the start of the measurement.

## Abbreviations

The following abbreviations are used in this manuscript:

MNPs	Magnetic nanoparticles
PEG	Polyethylene glycol
AMF	Alternating magnetic field
THP	Tumor-homing peptides
TNC-C	C-domain of tenascin-C
SQUID	Superconducting quantum interference device
