# Supplementary material for: Magnetic Detection of Cancer Cells Using Tumor-Homing Peptide-Modified Magnetic Nanoparticles
Source: Biosensors (Basel). 2026 Jan 5;16(1):45. doi: 10.3390/bios16010045 (PMC12838623; doi:10.3390/bios16010045)
Supplement: Supplementary file 1 [file biosensors-16-00045-s001.zip › biosensors-4049810-supplementary.pdf]

Supplementary Information for

Magnetic detection of cancer cells using tumor-homing peptide-modified magnetic nanoparticles

Shengli Zhou [1], Yuji Furutani [1], Kei Yamashita [1], Sakuya Kako [1], Kazunori Watanabe [1], Toshihiko Kiwa [1], Takashi Ohtsuki [1,*]

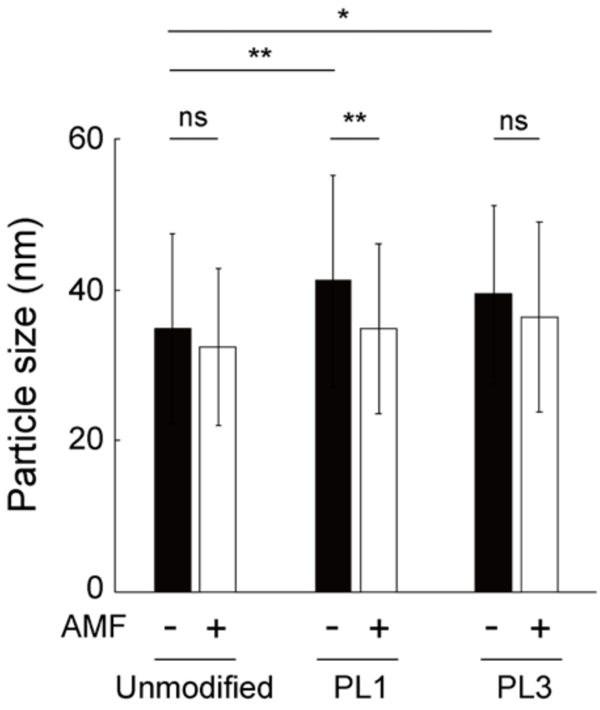

Figure S1. Average particle sizes (± SEM) of the magnetic nanoparticles (MNPs) before and after alternating magnetic field (AMF) irradiation, determined from transmission electron microscopy (TEM) images. More than 150 particles were measured for each MNPs. These results are expressed as means ± SEM. Statistical significance was analyzed using R [1] and EZR [2] software and determined using a two-way ANOVA with Tukey's test. *$p<0.01$, **$p<0.001$, ns: not significant.

[1] R_Core_Team, R: A language and environment for statistical computing. R Foundation for Statistical Computing. (2020).

[2] Y. Kanda, Investigation of the freely available easy-to-use software "EZR" for medical statistics, Bone Marrow Transplant. 48 (2013) 452–458. https://doi.org/10.1038/bmt.2012.244.

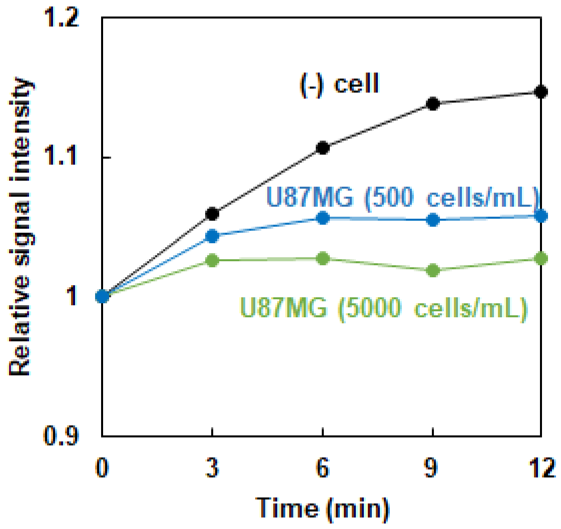

Figure S2. Magnetic signal intensities of PL3-MNPs that reacted with U87MG cell suspensions. The signal intensities were normalized to the intensity at the start of the measurement.
